# Gut microbiota: a non-target victim of pesticide-induced toxicity

**DOI:** 10.1080/19490976.2023.2187578

**Published:** 2023-03-15

**Authors:** Tusha Sharma, Nagabhishek Sirpu Natesh, Ramesh Pothuraju, Surinder K. Batra, Satyanarayana Rachagani

**Affiliations:** a Department of Biochemistry and Molecular Biology, University of Nebraska Medical Center, Omaha, NE, USA; b Department of Veterinary Medicine & Surgery, University of Missouri, Columbia, MO, USA; c Roy Blunt NextGen Precision Health Institute, University of Missouri, Columbia, MO, USA; d Fred & Pamela Buffett Cancer Center, Eppley Institute for Research in Cancer and Allied Diseases, University of Nebraska Medical Center, Omaha, NE, USA

**Keywords:** Gut microbiota, persistent organic pollutants, pesticides, organochlorine pesticides, organophosphate pesticides, heavy metals

## Abstract

The human gut microbiota can be potentially disrupted due to exposure of various environmental contaminants, including pesticides. These contaminants enter into non-target species in multiple ways and cause potential health risks. The gut microbiota-derived metabolites have a significant role in maintaining the host’s health by regulating metabolic homeostasis. An imbalance in this homeostasis can result in the development of various diseases and their pathogenesis. Pesticides have hazardous effects on the host’s gut microbiota, which is evident in a few recent studies. Therefore, there is an urgent need to explore the effect of pesticide on gut microbiota-mediated metabolic changes in the host, which may provide a better understanding of pesticide-induced toxicity. The present review summarizes the pesticide-induced effects on gut microbiota, which in turn, induces changes in the release of their secondary metabolites that could lead to various host health effects.

## 1. Introduction

Currently, there is an increased demand for agricultural production due to a significant increase in the growing population globally. Thus, the use of pesticides and fertilizers has become an impediment to meeting the increased demand for crops with the rise in population. However, the overuse of these pesticides increases the presence of pesticide residues in agricultural products, which is a severe risk to human health and the environment^1^. The widespread and inevitable use of pesticides threatens non-target species, including humans. Being the top consumer in the food chain, humans are the most affected non-target species by pesticide exposure. Various studies have reported pesticide levels in both human blood and urine, which are further found to be associated with multiple diseases, including cancer, diabetes, hormonal disorders, asthma, metabolic diseases, and neurotoxicity^2–5^. Furthermore, prolonged exposure to pesticides was also found to have teratogenic effects resulting in a reduction in birth weight, abnormal growth, diseases, and mortality^6,7^. Most available studies focused on the association between pesticide exposure and host health effects. Still, there is a paucity of studies focused on elucidating the mechanism involving the adverse impact of pesticides on human health.

In recent years, the research attention is shifted toward the effect of pesticide exposure on the host gut microbiota^8–11^. The host gut microbiota is a secondary endocrine system that maintains the host’s normal biological function through hormone secretion and immune regulation^12,13^. Altered gut microbiota may result in various diseases, including obesity, colon cancer, diabetes, and other metabolic diseases. It is also evident that pesticides could promote host metabolic disorders by disrupting gut microbiota function^8,10,14,15^. This review summarized the relationship between pesticide exposure, leading to alterations in the gut microbiota and their secondary metabolites release on host health effects, as well as the mechanism(s) by which different types of persistent organic pollutants induce changes in microbiota composition and function.

## 2. Gut microbiota

The ‘gut microbiota’ composed of bacteria, archaea, virus and eukarya, which are colonized in the Gastrointestinal (GI) tract^16^. They live as a complex community in the digestive tracts of humans and animals, as well as insects. It is estimated that in an average human lifetime, around 60 tons of food pass through the GI tract of humans, along with an abundance of microorganisms; many environmental factors like pesticides have a massive threat to gut integrity^17^.

### 2.1. Host gut microbiota and its metabolite

Around 10^14^ microorganisms with more than 500 species are present in the gut of adult humans, contributing to multiple biochemical and physiological activities of the host^18^. Gut microbiota comprises bacteria, archaea, viruses, and eukaryotic microbes, most of which are anaerobic microorganisms residing in the human GI tract^19^. In the gut of healthy adults human, *Firmicutes* and *Bacteroidetes* are the superabundant bacterial phylum of human gut microbiota, in addition to the *Proteobacteria, Verrucomicrobia, Actinomycetes, Fusobacteria, and Cyanobacteria*^18,20,21^. Gut microbiota has a significant role in the expansion and divergence of host intestinal epithelium, educating the host immune system, providing protection against pathogens, and maintaining intestinal homeostasis^22^. Various studies have demonstrated that gut microbiota regulates physiological function by releasing secondary metabolites^23^. The gut microbiota’s secondary metabolites include short-chain fatty acids (SCFAs), lipopolysaccharides (LPS), trimethylamine (TMA), branch-chained amino acids (BCAAs), bile acids (BAs), tryptophan, trimethylamine trioxide, and indoles^24^. LPS is one of the components released into the intestinal luminal environment upon bacterial death and lysis, which disrupts the host immune system and the blood coagulation system^25^. Due to the oxidation of flavin-containing monooxygenase 3 (Fmo3), TMA gets converted into trimethylamine oxide (TMAO), and is a critical risk factor for cardiovascular and cerebrovascular diseases^26^. BAs are the key player in the body’s energy metabolism by promoting digestion and absorption^27^. SCFAs are a chief fuel for intestinal epithelial cells and strengthen the gut barrier and immune-modulatory function via influencing GPR41/43^28^. Many indoles are produced by tryptophan metabolism, which maintains the intestinal barrier homeostasis and regulates the intestinal immune response^28^.

### 2.2. Regulatory mechanism of host gut microbiota

The function of gut microbiota is categorized into two aspects: 1. As a barrier in providing immunity and 2. to provide nutrition through metabolism. Additionally, gut microbiota also provides an indispensable barrier system to the body against pathogens via maintaining the steadiness of intestinal immunity^29^. Gut microbiota impedes the host immune system’s induction, training, and proper functioning of the host immune system. Under normal circumstances, gut microbiota composition is relatively stable until disturbed by a strong external intrusion. Once the composition of gut microbiota gets altered (dysbiosis), it may lead to endangering health conditions with an increase in the colonization of pathogenic bacteria^29–31^. Gut microbiota induces the activation of αβ-T cells by influencing immune pathway-related genes^32^. Further, it was also reported that gut microbiota regulates the inflammatory response via modulating TH17 cells in the small intestine^33–35^.

**Figure 1.**
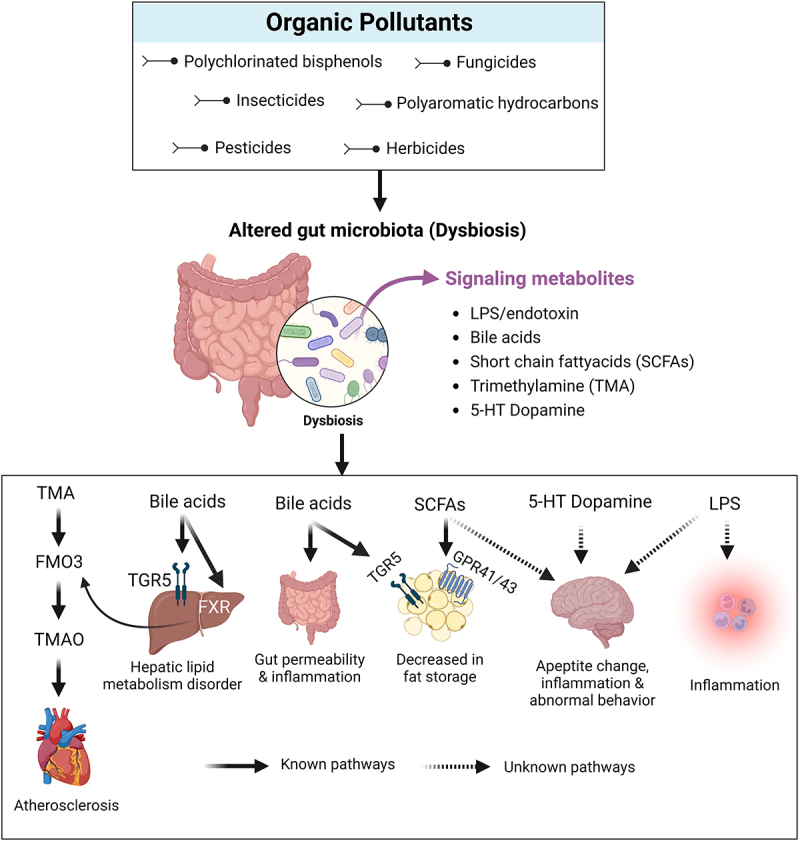
Graphical representation of the effect of pesticides and subsequent outcomes on gut microbiota. The figure represents different mechanisms that could alter gut microbiota composition and its metabolites induced via pesticide toxicity. The dysbiosis led by pesticide toxicity could adversely affect the host’s health through various known and unknown pathways.

In normal physiological conditions, the colon is mainly involved in water and mineral absorption. Further, the gut microbiota is vital in digesting undigestable fiber/carbohydrates to produce short-chain fatty acids, including butyrates. Due to this gut microbiota’s metabolic activity, the host’s epithelial cells obtain butyrate as an energy source required for growth and multiplication^36^. A study revealed that the composition of the gut microbiota of obese mice is entirely different from non-obese mice. However, there is a significant increase in the energy absorption capacity of the gut microbiota present in the host mice with obese conditions^36,37^. The SCFAs produced through fermentation of undigestable starch by the gut microbiota have significant importance on the host’s normal physiological function by entering peripheral circulation and providing more energy through metabolization^38,39^. SCFAs also affect the metabolism of fats, and carbohydrates, by activating the acetyl CoA synthase in the liver^39^. In a nutshell, the gut microbiota has a crucial role in controlling the function of the gut barrier, immune system, and host metabolic activity. In Figure 1, we have represented the host gut microbiota regulatory mechanism.

## 3. Role of persistent organic pollutants in the modulation of gut microbiota

Due to the lipophilic nature of persistent organic pollutants (POPs), these compounds are resistant to excrete out from the body once they enter into the body system^40^. Few POPs are naturally occurring, whereas most are man-made synthetic compounds for agricultural and industrial use. The accumulation of these compounds in the body system is hazardous to human health and is linked with immune dysfunction, reproductive and neurological disorders^41–44^. In recent years, harmful effects of POPs, which include polychlorinated biphenyl (PCB), polycyclic aromatic hydrocarbon (PAH), pesticides, insecticides, herbicides, and heavy metals, are recognized due to their effect on gut microbiota modulation and are extensively studied in terms of their toxic effects in human, and mice gut microbiota^40^ .

### 3.1. Polychlorinated (PCBs)

These compounds were banned in the United States three decades ago. However, PCBs are persisted in the environment and continue to contaminate food products. The primary route of exposure to PCB in the host biological system is through ingestion. The mice exposed to PCB showed a significant impact on gastrointestinal physiology and gut microbiota^45^. In addition, maternal exposure to PCB in mice significantly increased the gut permeability as well as the expression of gene Toll-like receptor 4 (Tlr4) and its signaling pathway in the colon of offspring^45^. *Bacteroidetes and Firmicutes* are two classes of beneficial bacteria in the human gut. Moreover, exposure to PCB increased the risk of developing obesity by altering the Firmicutes to Bacteroidetes ratio in mice and humans^12,46^. In addition, PCB exposure was associated with increased inflammation in the intestine in an age-dependent manner which is directly proportional to the abundance of proteobacteria^47,48^. However, more studies are required to conclude the mechanism behind PCB’s role on gut microbiota and ways to minimize these effects to decrease gut permeability and inflammation.

### 3.2. Polyaromatic hydrocarbons (PAH)

Polyaromatic hydrocarbons (PAH) gain less attention for their associated impact on gut microbiota than PCB. However, an *in vitro* study has shown that PAH exposure resulted in colonic microbiota transformation via acquiring estrogenic activity^47^. Also, microbial PAH transformation was confirmed by detecting its metabolite 1-hydroxypyrene and 7-hydroxybenzopyrene in the colon^49^. However, more studies will be needed to determine the possible effects of PAH on the gut microbiota.

### 3.3. Pesticides

Pesticide is a broad term which includes herbicides, insecticides, and fungicides. These pesticides are extensively used globally to increase crop production. These compounds are believed to be toxic to target species, but their residue on food products simultaneously affects non-target species, including humans, via an endocrine-disrupting mechanism^50^. Furthermore, recent studies have shown that the parent compound and their respective metabolites also cause adverse consequences to the environment and humans, especially the gut^15,51^. Recently, pesticide exposure and its effect on gut microbiota have gained more attention due to their increased use in crop production. Several studies concluded that pesticide exposure significantly alters the composition of gut microbiota, hence leading to a change in the metabolic profile of the host^1,52^.

#### 3.3.1. Organochlorine pesticides (OCPs)

Despite the ban on the usage of OCPs during the last three decades in several countries, their levels are continuously detected in the human body, which is a significant threat to human health^53,54^. Among all the OCPs, dichlorodiphenyltrichloroethane (DDT) and hexachlorocyclohexane are major compounds that can potentially metabolize to b-hexachlorocyclohexane and p’p’ dechlorodiphenyldichloro-ethylene (p’p’DDE), which are major breakout detrimental products in the environment. A recent finding has shown that serum OCP level in the general population is positively correlated with gut methanobacteriales and obesity^55,56^. Similarly, another study also demonstrated that the chronic exposure (8 weeks) of mice with β-HCH and DDT resulted in an alteration of the relative abundance and composition of gut microbiota by increasing *Bacteroidetes* and reducing *Proteobacteria*, *Deferribacteres, and Cyanobacteria* with increased quantity of the *Lactobacillus* strain with bile salt hydrolase activity. Significantly these alterations influenced the hepatic and bile acid hydrophobicity^27^.

Further, exposure of mice with β-HCH and DDT led to activation of *de novo* bile acid synthesis and inhibited the ileal bile acid reabsorption in mice. Findings from these studies are alarming and draw attention to the relationship of OCPs with the alterations in gut microbiota that need to be explored to illustrate further this relationship’s role in the pathogenesis of the disease^27,57^.

#### 3.3.2. Organophosphate pesticides (OPPs)

This category of insecticides are derived from amides and phosphoric acid. Due to their biodegradable nature, OPPs are the most frequently and commonly used insecticide worldwide. The residues of these compounds are found on plant surfaces, air, water, and soil, from which these compounds get leached out to the human system. OPPs residues in human plasma samples are found to be positively associated with diabetes. The gut microbial-mediated degradation of OPP alters the esterase activities and acetate, which ultimately induces gluconeogenesis and glucose intolerance^58^. Another study supports the findings and demonstrates that chronic exposure (180 days) of mice to OPPs induces glucose intolerance via the breakdown of OPP to acetic acid through the mechanism of gluconeogenesis by gut microbiota^58^.

The most extensively studied and widely used OPP is chlorpyrifos (CPF)^59^. Various *in vitro* and *in-vivo* studies revealed the ill effects of CPF^60–62^. The *in-vitro* model for the intestinal environment is the SHIME®, where the CPF exposure is found to be associated with decreased number of *Bifidobacterium* and *Lactobacillus* (beneficial bacteria; considered to be probiotics), accompanied by an increased number of *Enterococcus* and *Bacteroides*, which ultimately leads to the change in pH and stimulation of SCFAs^63–65^. Similarly, the effects of CPF on the Caco-2/TC7 cells were studied *in vitro*, and CPF could alter the mucosal barrier and cause inflammation through the divulging of IL-8. Although the above-mentioned *in vitro* models provide significant findings, there is a lack of microbiota and host crosstalk^64^.

Another study demonstrated that long-term exposure to CPF in mice resulted in increased gut absorbency and inhibited the expression of tight junction proteins, like claudin-1, ZO-1, and occludin in the epithelial lining of ileum and colon. Further, mice gut microbiota showed an increment in the Proteobacteria phylum and decreased Bacteroidetes phylum upon exposure to chlorpyrifos^15^.

Exposure to adult zebrafish with CPF resulted in alteration in the gut microbiota and metabolism^66^. It was observed that CPF alters the gut microbiota in rats by changing the abundance of bacteria associated with diabetes and obesity^67^. Whereas treatment of mice with a 1 mg/kg body weight dose of CPF for 30 days resulted in alterations in the gut microbiota with changes in urine metabolites, amino acids, metabolite, SCFAs, and bile acids elicited the intestinal inflammation and abnormal permeability of the intestine^52^. Further, treatment of pregnant rats until weaning with CPF revealed gut microbial dysbiosis in puppies, which can further result in impairment in the mucosal barrier (mucin 2) and an increment in bacterial translocation. Additionally, perinatal exposure of pups with CPF has a potential effect on the delayed maturation of intestines in pups and alters the development of the immune system^63,68^. Finally, CPF exposure decreases hepatic glucose and lipid metabolism via increased oxidative stress and microbiota dysbiosis in animal models^66^.

Diazinon is another OPP that raised public health concerns due to its extensive usage in agriculture. Its level is significantly traceable in drinking water, the primary route/source for human exposure to Diazinon^69–71^. Treatment of either sex mice with a four-ppm dose diazinon for 13 weeks caused gut microbiota dysbiosis, impaired energy metabolism, and activated multitudinous stress response pathways in male mice compared to female mice^72,73^. Exposure of rats for 14 days to diazinon led to induced histological changes and elevated the *Bacteroidetes, Firmicutes*, and *Fusobacteria* phyla in the gut of rats^74^. Malathion is one of the OPP known to alter gut metabolite (decrease taurine and glycine) and is also associated with increased inflammation^75^. All the above findings provide new insight into the relationship between gut microbiota and disease pathogenesis due to diazinon exposure.

#### 3.3.3. Herbicides

Herbicides are widely used as weed killers, but their residue on food grains like Maize/Soya bean potentially affects non-target species like humans causing harmful health effects. Likewise, traces of insecticides and herbicides are also detected in grains, fruits, and vegetables used for human consumption. Other significant sources of human exposure to these compounds through drinking water, air, and soil. Recently, several studies were directed to assess the impact of these compounds on gut microbiota and disease occurrence in various experimental models^76,77^.

##### 3.3.3.1. Glyphosate

Glyphosate-based compounds are extensively used as an herbicide in agriculture for controlling weeds^78,79^. Recent studies have illustrated that the effect of glyphosate on the gut environment was limited and is directly proportional to sufficient amounts of aromatic amino^80,81^. However, the detrimental effects of glyphosate were observed on the gut of individuals with malnutrition or consuming low protein diets. A recent study revealed a significant reduction of bacterial density upon the exposure of Hawaiian green turtles to glyphosate ≥ 2.2 × 10-4gL-1. Moreover, the abundance of *Pantoea, Proteus, Shigella*, and *Staphylococcus* has significantly reduced in a dose-dependent manner with glyphosate, indicating that glyphosate may have an adverse effect on gut microbiota and eventually on overall health^82^. Exposure of rats to glyphosate resulted in an alteration in the villi’s morphology in the duodenum and jejunum of rats.

Furthermore, glyphosate exposure suppressed the antioxidant mechanism by inducing inflammatory responses via increasing mRNA expression of IL-1β, IL-6, TNF-α, caspase-3, Mapk3, and NF-κB. Exposure of rats to glyphosate significantly decreased the abundance of Firmicutes compared to other phyla^82^. In contrast, another study demonstrated the deleterious effects of higher concentrations of glyphosate on immature bees through alteration in the richness of gut microbiota by shifting species diversity^83^. Most importantly, environmental exposure of human beings with glyphosate significantly affected the gut-brain barrier in humans by elevating the abundance of clostridium bacteria^84^.

##### 3.3.3.2. Pentachlorophenol (PCP)

Pentachlorophenol *(PCP) is* extensively used in paddy fields resulting in significant PCP contamination of the aquatic environment^85^. Interestingly, a study in goldfish demonstrated the change in the gut microbial community due to the toxic effects of PCP. In addition, PCP exposure to fish for 28 days revealed that the predisposition of PCP in the liver and intestine leads to growth inhibition, increased oxidative stress, and histopathological damage. Further, it also resulted in an altered ratio of *Bacteroidetes/Firmicutes* in the fish gut, causing the microbial community shift^77^. These findings increased interest in PCP-induced toxicity on gut microbiota dysbiosis and host health. Further, antibiotics significantly elevated the triazine-induced risks via gut microbiota dysbiosis^86^, supporting possible interaction between antibiotics, herbicide, and triazine. However, additional studies will be needed to establish the association between gut microbiota and human disease risk due to herbicide toxicity. Based on the available literature, the apparent effect of different pesticides on gut microbiota is collectively represented in Table 1.

**Table 1.** Impact of pesticides with the subsequent outcome on gut microbiota.

Categories	Specific Pesticides	Experimental Models	Outcome	Reference
Organophosphate pesticides	Chlorpyrifos	Mice and Wistar rats	Causes abnormal intestinal permeability, decreases Muc2 expression and alters the gut-brain communication and intestinal inflammation	^15,52,67^
	Atrazine	Zebrafish	Causes intestinal inflammation and oxidative stress	^109^
	Diazinon	Mice	Causes neurotoxicity through perturbation of gut microbiota	^72,73^
	Monocrotophos	Mice	Induces glucose intolerance	^58^
	Malathion	Mice	Induces pathogen invasion	^110^
Organochlorine Pesticides	β-Hexachlorocyclohexane	Mice	Altered gut microbiota composition and biliary bile acid profile	^57^
	p’p’-Dichlorodiphenyl dichloroethylene	Mice	Altered gut microbiota composition and biliary bile acid profile	^57,111^
Insecticides	Aldicarb	Mice	Increase pathogenicity of gut bacteria	^110^
Fungicides	Imazalil	Mice and Zebrafish	Cause colonic inflammation and also enhance microbiome diversity in the gut	^8,14,96^
	Carbendazim	Mice	Inflammation, a disorder associated with hepatic lipid metabolism	^9,14^
	Propamocarb	Mice	Disorder related to lipid and bile acid metabolism	^99^
	Permethrin	Wistar rats and Bacterial strains	Altered levels of SCFAs	^112^
	Epoxiconazole	Rat	Liver toxicity	^108^
Herbicides	Glyphosate	Swiss mice	Decrease intestinal bacterial count	^75^
	Pentachlorophenol	Goldfish	Hepatic histopathological damage	^76^

#### 3.3.4. Fungicides

This class of compounds is used to target and kill fungi and their spores. These biocidal compounds are generally fortified in post-harvested crops like fruits/vegetables and grains, causing significant contamination of agricultural products; consumption of this contaminated agricultural produce significantly resulted in health risks in humans and other animals, as discussed below.^87^

##### 3.3.4.1. Carbendazim (CBZ)

CBZ (methyl 2-benzimidazole carbamate) is a fungicide that inhibits fungal infections during agriculture storage and industrial processing^88^. However, CBZ potentiates disease pathogenesis by restricting liver oxidative stress, causing reproductive toxicity and endocrine disruption^89,90^. Oral high-dose administration of CBZ in mice for 28 days resulted in hepatic lipid metabolic disorder due to its accumulation in liver and fat tissue, eventually leading to inflammation^91^. Further, CBZ accumulates in the GI tract, where it interacts with gut microbiota leading to dysbiosis due to decreased population of *Bacteroides* and an increased abundance of *Firmicutes, Proteobacteria, and Actinobacteria*^9^. Further, chronic exposure of mice with CBZ for 14 weeks resulted in dysbiosis of gut microbiota leading to dyslipidemia and facilitated the intestinal absorption of triglycerides leading to increased inflammatory response^92^. Therefore, CBZ exposure potentially alters the gut microbiota and affects overall health by disrupting host hepatic lipid metabolism and inflammatory response in the host.

##### 3.3.4.2. Imazalil (IMZ)

Like other fungicides, IMZ is a well-known compound used to protect post-harvested crop produce and is often detected in soil, water, fruits, and vegetables^93–95^. Exposure of zebrafish with IMZ at the early development stage potentially induced developmental toxicity and locomotor behavior abnormalities^96^. Further, exposure of zebrafish with IMZ for 21 days resulted in dysbiosis and altered hepatic metabolism^8^. Additionally, exposure of mice with IMZ for 28 days (100 mg/kg body wt/day) potentially initiated mouse colonic inflammation and gut microbiota dysbiosis via decreasing abundance of *Firmicutes* and increasing *Proteobacteria, Actinobacteria, and Bacteroidetes*. Simultaneously, IMZ exposure significantly reduced the *Lactobacillus* population and *Bifidobacterium, whereas* elevated *Deltaproteobacteria* and *Desulfovibrio*^92^. Chronic exposure of mice with IMZ at a low dose altered the intestinal barrier function in mice. These results also revealed that IMZ exposure is associated with reduced mucosal release and downregulates the expression of cystic fibrosis transmembrane conductance regulator in the ileum and colon of the mice^97^.

##### 3.3.4.3. Propamocarb (PM)

PM in the environment is another health threat to the human population. It is known as carbamate fungicide, which controls diseases associated with the Oomycetes^98^. The residue of PM was detected in humans and shown to have detrimental effects on health^99^. Hepatic lipid metabolism was perturbed in zebrafish upon high-dose exposure to PM for a short duration^11^. Previous studies have shown that PM exposure resulted in perturbation of the microbial metabolite due to gut microbiota alterations. Exposure to PM at a dose of 300 mg/L for four weeks led to dysbiosis, which resulted in altered fecal metabolites, TMA, SCFAs, and succinates, leading to detrimental effects on the host’s health^100^. TMA is the key molecule involved in the development of atherosclerosis, which is significantly elevated in the feces upon exposure to PM.

Further, the PM also alters the cardiac NO/NOS pathway and upregulates NF-κB at the transcriptional level, ultimately increasing the risk of cardiovascular disease through induction of the enterohepatic metabolism^99,100^. Moreover, the composition of gut microbiota and fecal metabolites involved in energy metabolism in mice was significantly altered due to long-term exposure to a low dose of PM. Nevertheless, gut microbiota is reported to metabolize dietary choline to TMA, which is further oxidized to TMAO in the liver by flavin monooxygenase-3 and the farnesoid receptor. The increased level of TMAO is a potent biomarker for the risk assessment for the development of cardiac diseases^101–103^.

##### 3.3.4.4. Epoxiconazole

This category of fungicides belongs to the azole group and use to intercept the diseases like leaf blotch and rust in wheat^104,105^. Therefore, consuming contaminated wheat products was a potential source of exposure to the human population. Furthermore, the previous study indicates that exposure of female rats to different doses of epoxiconazole (4 mg/Kg/day and 100 mg/Kg/day) for 90 days potentially modified the composition of the gut microbiota in rats by decreasing the level of Firmicutes and increasing the abundance of Bacteroidetes and Proteobacteria. This alteration in the abundance of Bacteroidaceae (increase/decrease) is associated with pouchitis and Crohn’s disease. Similarly, the *Lactobacillaceae* known to protect against liver damage is found to be influenced due to the exposure of epoxiconazole^106–108^. All these studies have shown the potential effect of epoxiconazole in altering gut microbiota and inducing hepatic toxicity, suggesting that early monitoring could be a better preventive measure to avoid potential health risks to the host^109^. To summarize, fungicides are potent compounds affecting human health by modulating gut microbiota. However, further mechanistic-based studies would be needed to fulfill the significant gap and the scope of research to explore fungicides direct impact on gut microbiota.

#### 3.3.5. Insecticides

Insecticides are potentially used to increase crop production; however, their chronic application on crops leads to their residue/remnants on agricultural commodities resulting in harmful effects on the health of humans and other animals^110,111^. As evident from the available literature, these compounds, including imidacloprid and thiamethoxam, are toxic to the environment and accumulate in the food chain, which is one of the significant concerns to human health. Their levels are continuously detected in the human body fluids vary significantly, indicating that these compounds enter the human body leading to a predisposition to different diseases^112–114^. The gut is one of the significant non-target organs majorly affected due to the exposure of insecticides directly or indirectly through contaminated food and water, which gains the researcher’s attention to identify the toxic effect of insecticides on gut microbiota and their consequences on human health.

## 4. Role of pesticide exposure in modulating host health via gut microbiota

The harmful effects of pesticide contamination on non-target species have increasingly gained more attention in the last few years. Surprisingly, few researchers have identified the function of gut microbiota in the detoxifying pesticide effects to maintain the host metabolic homeostasis^1,83,115^. Dysregulation of gut microbiota composition is found to be associated with metabolic disorders, which can further lead to diseases like diabetes, colon cancer, etc.^116,117^. Various studies have unraveled the mechanism of pesticide toxicity on non-target species. The usage of various organochlorine pesticides, including β-HCH and p’p’ DDE were banned three decades back. However, their residual levels are still detected in the environment at a significant level^53^. Alteration in metabolites, diversity changes, and energy interference are the three indicators of microbiota disturbance and may impact host health via increasing disease risk. Therefore, the toxicity assessment of gut microbiota can potentially bridge environmental exposure and human health/diseases. Thus, this section discusses the connection between gut microbiota toxicity and human health. Pesticides and other xenobiotics are known to influence the physiologic function of the gut microbiota and are responsible for the pathogenesis of various diseases (Table 2). Consistent modulation in the gut microbiota due to exposure to a particular chemical could be a potential biomarker to assess its toxicity. SCFAs, one of the metabolites of gut microbiota, acts as a signaling molecule that binds to cellular receptors, including G-protein coupled receptor (GPCRs) and transmembrane G protein-coupled receptor 5 (TGR5). Farnesoid X receptor (FXR)^118^ activates the signaling cascade, contributing to regulating metabolic activities through the gut-brain barrier. SCFA and bile acids affect insulin secretion and glucose homeostasis, possibly leading to the risk of developing diabetes^119,120^. The tryptophan metabolites can contribute to the modulation of intestinal immune cells and barrier functions via activating aryl hydrocarbon receptor (AHR), which is involved in the inflammatory bowel disease (IBD) pathogenesis ^121–123^. Gut bacteria convert choline and L-carnitine into trimethylamine, which is metabolized in the liver in triethylamine N-oxide (TMAO), which is significantly associated with cardiovascular disease risk^124,125^. Fermented products of proteins like polyamines and N-nitroso compounds derived by gut bacteria deploy carcinogenic effects and promote colorectal malignancy^125,126^. Environmental chemicals like diazinon and nicotine perturb bacterial metabolites that are neurotransmitters (serotonin, gamma-aminobutyric acid), resulting in neurotoxic effects^127^. Exposure of mice to various pesticides, especially organochlorine compounds β-HCH and DDE, could hinder the constitution of gut microbiota leading to further disturbance in the metabolism of bile acids and simultaneously decreasing the β–Muricholic acid (β-MCA), which is an important component that regulates the metabolism of bile, lipid, and glucose^57^. There is an alleviation in high-fat diet-induced obesity in mice upon exposure to endosulfan sulfate, resulting in alteration in gut microbiota, lipid metabolism, and glucose homeostasis^128^. Apart from organochlorine pesticides, chlorpyrifos, an organophosphate pesticide, has been shown to possess significant toxic effects on the gut microbiota. Chronic exposure to chlorpyrifos may lead to inflammation in the intestine via influencing gut microbiota, disturbing the urine and liver metabolism, and increasing the mucous volume in the intestine, which leads to the predisposition of oxidative stress^52,66^. Other pesticides like nitenpyram and imazalil are known to impact gut microbiota negatively. Prenatal exposure of mice with nitenpyram resulted in dysregulation of gut microbiota and fecal metabolites like purines, BCAAs, and TCA cycle components leading to elevated consumption of the host energy^128^. To assess the toxic effect of imazalil, the zebrafish model was used, and it was found to alter the gut microbiota and influence liver metabolism^8^. Various pathways, including the TCA cycle, amino acid/lipid/nucleotide, and glucose metabolism, were also altered after exposure to imazalil^8^. Similarly, short-term exposure to pro-paraben and propamocarb resulted in adverse effects on the gut microbiota and liver metabolic profile^129^. It was observed that there are approximately 20 fecal metabolites were affected due to the exposure to the propamocarb^99^. Previous studies also revealed the impact of exposure to penconazole on gut microbiota and serum metabolic profile in mice^130^. Exposure to pesticides can significantly impact the gut microbiota, leading to an imbalance of microbiota composition, which may further cause metabolic diseases in the host. However, in the future, more studies will be required for a further and in-depth understanding of mechanisms and their effect on the host.

**Table 2.** Gut microbiota toxicity associated with specific pesticide and heavy metal exposure.

Category	Compound	Effect on Gut Metabolite	Effect on Gene/Pathway	Adverse Outcome
Heavy Metals	Arsenic	Decrease Indole Lactic acid, Daidzein and glycocholic acid	Increase LPS, DNA repair, and multidrug resistance	Inflammation^128,136–138^
	Cadmium Lead	Increase LPS and decrease Vitamin E and bile acids	Increase oxidative stress and phenylalanine synthesis	
	Manganese	Decrease Vitamin E and increase phenylalanine	Decrease phenylalanine synthesis	
Pesticides	Carbamate aldicarb	Increase 1-Methylnicotinamide	Increase virulence, adhesion, and bacteriocins	Inflammation^75,76,90,93,97^
	Carbendazim			
	Diazinon	Decrease Taurine and glycine	Increase Tryptophan	
	Imazalil			
	Malathion		Increase Quorum sensing, virulence, and pathogenicity	

## 5. Other potential environmental contaminants

Heavy metals are one of the extensively studied environmental contaminants, but their role in gut toxicity is unidentified. The gut bacterial flora is vital in the biotransformation of heavy metals via promoting or attenuating their toxic effects. For example, arsenic exposure is one of the major concerns to human health through the ingestion of contaminated water. A study has shown that human gut microbiota can convert inorganic arsenic into an organic form that is less toxic to human health^131^. Exposure of mice to arsenic, cadmium, cobalt, chromium, and nickel in drinking water resulted in alterations in the gut metabolic profile^132,133^. In agreement with this finding, another study also showed the alteration in gut microbiota after exposure to a 100 ppb dose of arsenic in mice^134^, which provides a new horizon to evaluate the pathways behind arsenic-induced toxic effects in humans. Further, the arsenic exposure in the Bangladesh population significantly modulated the gut microbiota by promoting an increased abundance of *Citrobacter*, which is responsible for various health concerns in humans, including urinary tract infections, respiratory diseases, inflamed gastrointestinal tract, cardiovascular disease, specifically atherosclerosis through TMAO, etc.^135–137^. Previous studies have shown that exposure to heavy metals like arsenic, cadmium, lead, and manganese may contribute toward the inflammatory events in the gut via a decrease in indole lactic acid, daidzein, and glycocholic acid, vitamin E leading to an increase in LPS^138–140^ level in the lumen of the gut. Gut microbiota also performs the demethylation of mercury and its conversion into methylmercury, a more toxic form of mercury^141^. Mercury is another heavy metal extensively studied for its effect on the microbiota in several experimental models, including mice, rats, chickens, fish, and humans. Exposure to methylmercury antedate damages the GI tract and alters the gut microbiota^142,143^. However, there are studies conducted to examine the effects of heavy metals on gut microbiota composition (Table 2). Still, there is a paucity of literature that explores the mechanism behind the heavy metal-induced toxicity of gut microbiota. In the future, there is an urgent need to conduct more mechanistic studies to explore heavy metals’ toxic effects on gut microbiota and metabolic alteration.

## 6. Future perspectives and conclusion

In recent years, due to the extensive usage of pesticides globally, their harmful effect on human health has gained significant attention. To avoid the detrimental impact of pesticides on human health, biopesticides could be a promising replacement. This could include microorganisms that target pathogens or RNA-based biopesticides. The use of the synthetic biology approach may offer a better future for agriculture with minimal harm to human health. All the available studies have correlated to pesticide exposure leading to alterations in host gut microbiota composition and metabolic profiles. However, gut microbiota toxicity has yet to be explored so far due to a lack of literature on mechanistic-based studies.

Further, the available studies have yet to provide a concrete relationship between a particular pesticide and microbiota or metabolite to assess its toxicity. Therefore, there is vast space to explore the mechanism behind the toxicity induced by pesticide exposure to gut microbiota and associated metabolites. In the future, deeper sequencing techniques should be adopted, like shotgun sequencing, as well as 16s RNA will be required to identify the strain and species, which are altered explicitly due to pesticide exposure in the gut.

**Figure 2.**
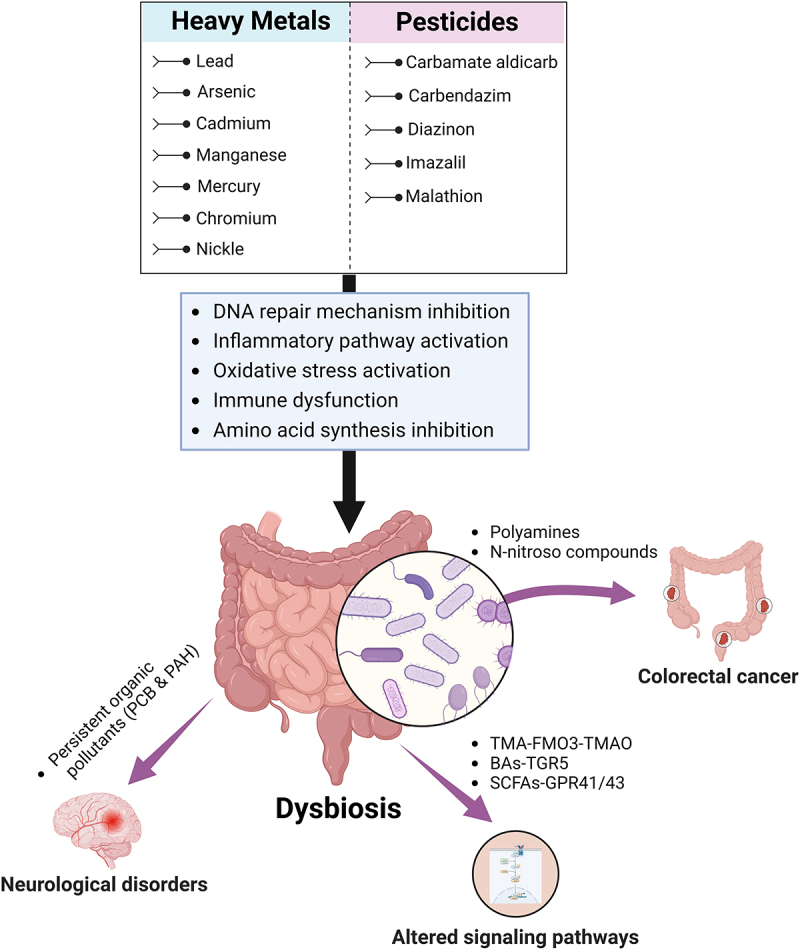
Effect of the predisposition of different environmental contaminants (pesticides and heavy metals) on the functional changes of gut microbiota of the host. The functional modification in gut microbiota leads to diversity loss and alteration in functional metabolites. This subsequently results in the pathogenesis of various diseases, including colorectal cancer, via invasion of IBD pathogens and accumulation of non-digestible carbohydrates.

To conclude, pesticide exposure may induce toxicity to gut microbiota by modulating gut microbiota composition and their functional changes (summarized in Figure 2). Due to these changes, gut microbiota can be a potential biomarker to assess pesticide toxicity. Furthermore, pesticide exposure is linked to various human diseases due to diversity loss, alteration in metabolic profiles, and energy metabolism. Although there is multiple evidence for the effect of environmental exposure on the gut microbiota, it is propitious to evaluate the mechanism of such disturbance through the lens of toxicology. This review emphasized pesticide toxicity on gut microbiota and xenobiotic-induced damage to human health and disease. Further development of biomarkers development by assessment and modification in gut microbiota composition. Thus, an overall in-depth evaluation of xenobiotic-induced toxicity on the gut microbiota will provide insight into environmental exposure and human gut health, which may facilitate the development of biomarkers for therapeutic interventions.

## Abbreviations

PCP: Pentachlorophenol
CPF: chlorpyrifos
HCH: b-hexachlorohexane
DDT: p’p’ dichlorodiphenyltrichloroethane
p’p’DDE: p’p’ dechlorodiphenyldichloro-ethylene
HCH: hexachlorocyclohexane
OCPs: Organochlorines pesticides
Tlr4: Toll-like receptor 4
PAH: polycyclic aromatic hydrocarbon
PCB: polychlorinated biphenyl
POPs: persistent organic pollutants
TMAO: trimethylamine oxide
Fmo3: flavin-containing monooxygenase 3
LPS: lipopolysaccharides
TMA: trimethylamine
SCFAs: Short-chain fatty acids
BA: bile acids
PCP: Pentachlorophenol
CBZ: methyl 2-benzimidazolecarbamate
IMZ: Imazalil
CFTR: cystic fibrosis transmembrane conductance regulator
PM: Propamocarb
FXR: farnesoid X receptor
GPCRs: G-protein coupled receptors
AHR: aryl hydrocarbon receptor
IBD: inflammatory bowel disease
β-MCA: β – Muricholic acid
